# Assessing the Impact of Green Hiring on Sustainable Performance: Mediating Role of Green Performance Management and Compensation

**DOI:** 10.3390/ijerph18115654

**Published:** 2021-05-25

**Authors:** José Moleiro Martins, Hira Aftab, Mário Nuno Mata, Muhammad Ussama Majeed, Sumaira Aslam, Anabela Batista Correia, Pedro Neves Mata

**Affiliations:** 1 ISCAL-Instituto Superior de Contabilidade e Administração de Lisboa, Instituto Politécnico de Lisboa, Avenida Miguel Bombarda 20, 1069-035 Lisbon, Portugal; mnmata@iscal.ipl.pt (M.N.M.); ambatista@iscal.ipl.pt (A.B.C.); 2 Instituto Universitário de Lisboa (ISCTE-IUL), Business Research Unit (BRU-IUL), 1649-026 Lisboa, Portugal; 3 Institute of Business and Information Technology, University of the Punjab, Lahore 54590, Pakistan; usamamajeed845@gmail.com (M.U.M.); sumeraaslam18@gmail.com (S.A.); 4 Polytechnic Institute of Santarém, School of Management and Technology (ESGTS-IPS), 2001-904 Santarém, Portugal; 5 ISTA-School of Technologies and Architecture, Instituto Universitário de Lisboa (ISCTE-IUL), ISTAR-IUL, Avenida das Forças Armadas, 1649-026 Lisboa, Portugal; pedronmata@gmail.com

**Keywords:** green human resource management, sustainability, environmental performance, green hiring, green performance management, and compensation

## Abstract

The global need to preserve ecology has propelled the green movement across the globe. An emerging managerial challenge for all organizations is to protect natural resources by reducing their negative impact on the environment and increase sustainable performance. Greening is the need of the age to conserve natural resources. This study investigates the impact of green human resource management practice—i.e., green hiring—on the sustainable performance of public and private healthcare organizations. A quantitative research approach was used for data collection. Scale survey of 160 responses was gathered from public and private healthcare organizations. Partial least square–structural equation modeling was used for data analysis. The study results suggest that green recruitment has a positive and significant impact on environmental performance, economic performance, and social performance. Path coefficients test also revealed that green performance management and compensation significantly mediate the relationship between green hiring and sustainable performance of public and private healthcare organizations. This study is helpful for organizations in adapting GHRM practices that will benefit the organizations in all ways. This study also provides a better understanding to policymakers on how to promote GHRM practices and increase sustainability in organizations.

## 1. Introduction

Working to preserve the environment, protecting resources, and reducing exploitation of natural reserves is critical for today’s world. The green movement across the globe has given rise to the concept of ‘green human resource management’ (GHRM) practices. Excess consumption, exploitation, and deterioration of natural resources harm the greening of the organization [1]. Issues like global warming, hiking levels of pollution, carbon profiles, climatic changes, flooding, extinction, and endangering of species make it obligatory for all the sectors of the economy to put in efforts to preserve the environment and work for the welfare of society [2]. The constant warning from scientists and environmentalists regarding the ecological conditions had created a serious situation for industries and organizations across the world. We need to opt for strategies that conserve the natural environment [3].

Proper integration of environmental innovation of the firm with its internal capabilities is necessary to survive in a competitive industrial setting. The development of green strategies, along with new competitive tools, can help organizations to increase the environmental performance of the organizations [4]. Product and service markets across all sectors need to look into this matter as environmental issues eventually lead to causing harm to all [5]. The increasing population is leading towards more pollution from industrial activities. Therefore, governments, stakeholders, customers, employees, and competitors are putting pressure on firms to adopt environmentally friendly practices in the long run. These practices can help firms for better sustainable performance and to gain a competitive advantage [2]. Customers prefer those organizations that work for the welfare of society and reduce negative impacts on the natural environment. Green organizations develop good CSR practices and work for the protection of the natural environment, green culture, and a better economy [6].

Human resources, the workforce, or employees are the most critical asset of the organization and are responsible for bringing any changes in firms. Human resource management (HRM) is the essential activity that handles the employees, trains them so that they perform their activities in a better way. GHRM is a new alternative to traditional HRM. GHRM practices, policies, and frameworks create a green culture, and help in the sustainability and preservation of the environment [7].

Recent research is conducted to explore the potential impact of GHRM and employees’ environmental performance in different sectors of a country. Research conducted in the IT industry of developing countries revealed that organizations not only reduce carbon emissions through adopting GHRM practices but also lead to cost savings. These practices help to conserve energy and improve the profitability of the organization [8]. It has also been revealed that a better economy, reduction in costs, and efficient usage of resources can be promoted through the implementation of GHRM strategies. Other past research is conducted to study the impact of GHRM practices on the performance of firms of a developing country—i.e., Pakistan. Findings of this past study revealed that green practices are of vital importance for firms to perform well and achieve sustainable economic performance [9].

Every sector of the world is adopting environmentally friendly practices, but this trend is less prevalent in developing countries. Many of the developing countries are not adopting green practices fully in their different functional areas [10]. A few studies were conducted to analyze the implementation of GHRM practices in the service sector of developing countries. Therefore, the current study explores the impact of green hiring on the sustainable performance of the service sector of a developing country. It is an essential contribution of the current study. The second key contribution of the current study relates to the mediating role of green performance management and compensation between green hiring and sustainable performance of public as well as private healthcare organizations. This is the major contribution of this study as no past research has been conducted to study the mediating effect of green performance management and compensation on the relationship between green hiring and sustainable performance. Third, a conceptual framework of the current study is providing information to healthcare organizations to improve the social, economic, and environmental performance of firms by applying green strategies. Moreover, it would help to handle the governmental associations, customers, competitors, and stakeholders’ pressure.

### Research Objectives and Questions

Organizations are continuously trying to minimize the negative impact created by them on the environment and at the same time try to improve their sustainable performance. The healthcare service industry is in a difficult situation due to the global COVID-19 pandemic, making this research more relatable to explore the impact of green hiring on the sustainable performance of a particular sector, i.e., the healthcare sector. In past research, it is explored that the organizations that follow the process of green recruitment attract more and well-qualified candidates [11]. It is very important to assess that employees are hired based on necessary skills and more knowledge of environmental aspects can help firms to attain sustainable performance. Therefore, this research aims to analyze the concept of green hiring and the impact it creates on the environmental, social, and economic performance of the public, as well as private, healthcare organizations. Based on these discussions, the first research objective of this study is proposed below. Research objectives (RO) and research questions (RQ) of the study are as follows:

RO1. To investigate that green hiring has an impact on the sustainable performance of healthcare organizations.
RQ1. Does green hiring have an impact on the environmental performance, economic performance, and social performance of health care organizations?

Green performance management and compensation have significant importance as employees are punished and rewarded based on their adherence to the environmental values followed in many sectors. In past research, it is depicted that the organizations give rewards, appraisals, and do evaluations based on the adherence to green standards and practices leading to survival, sustainability, and also creating a positive image [12]. To guide this evaluation, the current study aims to investigate the mediating role of green human performance and compensation on the green hiring–sustainable performance relationship. Based on this discussion, the following is another research objective:

RO2. To investigate the mediating role of green performance management and compensation on green hiring–sustainable performance relationship
RQ2. Does green performance management and compensation mediate the relationship between green hiring and environmental performance?
RQ3. Does green performance management and compensation mediate the relationship between green hiring and economic performance?
RQ4. Does green performance management and compensation mediate the relationship between green hiring and social performance?

The latest tool and techniques are used to answer the research questions. Primary data is collected from HR and non-HR staff of the hospitals by the detailed and comprehensive research questionnaire; used to measure green hiring and sustainability performance of the hospitals. The most widely used software, Smart PLS 3.1, is used in this research to analyze the quantitative data. Both structural and measurement models are analyzed with the help of PLS-Structural Equation Modeling.

## 2. Literature Review and Hypothesis Development

The leading challenge around the globe is achieving a balance between sustainable development and economic growth and progress. The increase in population and the need for a sustainable future requires efforts to preserve the resources and environment [13]. There is a need to adopt such practices that promote sustainability and help in preserving the resources for the next generations [14]. GHRM is one popular concept that has attracted many people across the globe. It is famous around the world, but it has different meanings for different people [15]. Effective environmental management is an urgent need across the world. Damage caused by pollution, several harmful pollutants, and industrial waste have majorly contributed to the deterioration and depletion of valuable natural resources [16]. It is swift and evident across the globe. It is necessary to preserve and improve the environment for the current and coming generations [17]. To achieve economic development without causing significant damage to the ecology, organizations must work on environmental management practices [18].

It is necessary to work on the significant impact of the organizations on environmental competitiveness. GHRM is a powerful force in creating a workforce that makes, appreciates, and understands the green culture of the organization [19]. The process of human resources needs to be aligned with sustainability and the natural environment to achieve their goals. Green initiatives are to be incorporated in all human resources activities such as hiring, training, and compensation to create a sustainable culture within organizations [20]. The development of a green culture and incorporating it into the inner culture of an organization helps in building specific values in that organization [21]. Green practices help an organization in reaping more profits and leading it to greater financial savings. These practices, if followed, benefit the natural system and provide the employees with a productive workplace in a socially sustainable way. It refers to the commitment and involvement of every employee within the organization to contribute to the organization’s sustainability [22]. It is the use of green practices to resolve the concerns of people management and policies in attaining a broader corporate schedule of the environment [23].

Green hiring, which is often called green recruitment, is hiring employees with the necessary skills, knowledge, approach, and behaviors to identify environmental management systems [24]. Nowadays, many organizations have understood the need for green hiring, which also builds branding. Green hiring is considered as one of the essential dimensions in GHRM that enabled the organization to attract a pool of environmentally responsible candidates by focusing on environmental knowledge and motivation. Moreover, it led to the creation of a green workforce that successfully and effectively contributed to the ecological practices of the organization [25]. Organizations must incorporate green hiring practices to improve performance and comply with ethical standards. Green hiring has been linked to the reinforcement of the environmental practices that lead to sustainability and increase employees’ organizational commitment. It is the application of environmental management in the human resource management function of the organization to improve its efficiencies and lead to better environmental performance [26].

Green training and employee engagement are the critical aspects of the green hiring process, which should be implemented with proper attention. Most employees tend to resist new green policies and practices implemented as it hinders their ability to use their knowledge and stick to the additional duties under the process [27]. However, green training assists in improving the performance of the organization. It helps the organization in enhancing the competencies of the employers in the green cause. It makes the employees to get satisfied with their jobs [28]. The involvement of the employees in green activities is an important factor in the green hiring process. Employees hired based on their skills and knowledge about environmental aspects will help the organization to achieve environmental sustainability. It fosters the individual performance of the employees. Moreover, green training also enhances the green creativity of the employees [29].

Green training educates the employees about the importance of environmental management, helps them conserve energy, reduce waste, diffuse awareness about the environment in the organization, and engages employees in ecological problem-solving [30]. Moreover, it also enhances the employees’ understanding of environmental management. When combined with an excellent ecological culture, green training and development involve the employees towards environmental goals [31]. It is suggested that green training enables the organization to achieve eco-friendly managers who indulge in environmentally friendly activities and promote sustainability [32]. It implies that the organizations should not only train their employees according to best business practices with green initiatives but should also encourage the customers to become more environmentally friendly and promote the practice of buying green products [33].

GHRM is one of the essential factors in analyzing the sustainability of the organization. Maintaining the sustainability of the world’s ecosystem in this era of the ever-changing environment with continuous services for human beings is the prime focus of the managers of this age [34]. Firms today are becoming more aware of their social, ethical, and ecological perspective; they are promoting sustainability and sustainable goals [35]. GHRM is one of the necessary long-term goals of any organization. Achieving sustainable goals helps the organization to become socially and economically efficient. The importance of sustainability in human resource management has two aspects: one in managing people and the second in the notion of sustainable performance. Valuable and practical human resource management is critical for success related to organizational performance [36]. Secondly, sustainability is not a minor factor; it is necessary for the long-term survival of the organization, and it is considered a strategic and critical element of the process of green hiring [37].

Sustainable development requires a balance from the organization in every internal and external factor, keeping the short-term and long-term perspectives in view. There is a need to manage the organization’s social, environmental, and economic well-being to achieve sustainability [38]. Sustainability practices need to be incorporated in every facet of the organization and its strategic management process, with a critical focus on green hiring and green performance management, as it is the dominant factor in raising the employees towards sustainability, and it also improves the human capital management process [12].

The healthcare sector is considered to be responsible for harmful emissions that adversely affect health. Healthcare sustainability science raised the need for different tools and metrics to control resource consumption and harmful emissions, leading to improved patient health and the protection of the environment [39]. A transformation in healthcare organizations is required to enhance the services, safety, quality, and value of these organizations [40]. The transition of healthcare is a global concern that tends to improve the sector economically, socially, and environmentally. Instead of short-term focus, long-term perspective must be opted to minimize the inevitable loss [41]. The green building concept was also proposed to design the healthcare buildings using the green concept as a strategy to overcome the ever-growing global warming challenge [42]. Along with the already existing practices like waste management, green building, and unique product designs, there is the need for governmental and non-governmental organizations to provide peer support to educate employees about green healthcare for best practices and continuous improvement in the field [43].

Organizations can enhance the green image and environmental performance by developing a reputation as a green employer [44]. Nowadays, companies are linking job roles and job tasks with environmental issues. These job roles and job descriptions are advertised to acquire individuals that are more focused on environmental performance [45]. Moreover, companies are more focused to hire job seekers having pro-environmental behaviors [46]. Environmental performance depends on green hiring, green training of employees, assessment of the organization’s environmental goals, and green culture [47]. Pro-environmental behavior and green practices have a significant impact on the improvement of environmental performance in an organization [48]. Similarly, employees’ involvement and awareness about environmental goals tend to increase sustainable practices at the workplace and provide support to management to achieve environmental performance [49]. The green behavior of the employees leads to human well-being, social betterment and consequently fulfills the environmental goals of sustainability [10]. Understanding and implementing green culture can assist any company with determining that employees are perpetrated towards green practices and objectives of the environmental performance [50]. Therefore, this study suggests that:

**Hypothesis** **1** **(H1).** *Green hiring has a positive and significant impact on environmental performance*.

A recent study on the behaviors of employees and job satisfaction in the Italian healthcare sector suggested that green training is beneficial for improving job satisfaction and pro-environment behavior of the employees. It is also revealed that green hiring enhances the employee’s competencies to revamp the organization’s economic performance. Eventually, the profits of the organization can be increased, and an improved society can be established [30]. Every organization performs environmental management activities to achieve ecological goals. Employees play a significant role in the corporate environmental management of the organization. To enhance the organization’s ecological, social, and economic performance, the right person should be hired through green hiring [51]. There are stakeholders and competitors’ pressure to balance the environmental and economic performance to survive in the market [52]. Similarly, there is a positive association between economic performance, environmental management, environmental performance, and green human resource practices including green hiring and training [53]. Similarly, there is a positive relationship between green human resource strategies and the economic performance of the organization. Alignment of green human resource strategies with sustainability concepts will provide ease to the firms to achieve short-term financial objectives and as well as the future goals of sustainability [54]. Moreover, hiring a workforce that is committed to environmental development will eventually help the firm to achieve sustainable goals. Similarly, excellent training and employee’s commitment and involvement in environmental issues may enhance their skills and knowledge, thus improving the economic performance [55]. Based on these discussions, the following hypothesis evaluates the positive relationship between green hiring and economic performance.

**Hypothesis** **2** **(H2).** *Green hiring has a positive and significant impact on economic performance.*

The three pillars of sustainability refer to people, profits, and the planet. At the same time, the most attention is given to the environmental factor, which impacts food production, carbon footprint, wastage, and other externalities which ultimately lead to environmental issues. The need for the company to survive globally successful in the local climate makes the social pillar most significant. Lastly, due to the economic pillar, the organizations can implement sustainability strategies [56]. Firms should have to consider their corporate social responsibility strategies while attracting or hiring a workforce, to escalate organizational image among the society [57]. Similarly, there is a positive relationship between green human resource practices and CSR. Eventually, it will reflect an environmentally and socially responsible image of the firm. Thus, green HRM strategies have a positive impact on the environment and society [58]. Based on these discussions, the following hypothesis is formulated:

**Hypothesis** **3** **(H3).** *Green hiring has a positive and significant impact on social performance.*

In addition to other factors proposed, green hiring and green performance management and compensation have played a vital role in greening healthcare centers and transforming the traditional sector towards a sustainable one [59]. Human resource management plays a vital role in developing sustainability throughout the organizations. The organizations must expand their strategies and build a sense of greening in the employees [60]. Industries can achieve better environmental, social, and economic performances through adopting the practice of green hiring. It is the need of the hour that the organizations have to develop a plan to educate their employees, motivate them because enthusiastic participation from the employees has to be increased to achieve greening of the organization [61]. Organizations need to put the environment as a central plan and motivate the workforce to opt and adapt the greening. Green performance management and compensation also encourage the employees to participate in environmentally friendly tasks [62]. Essentially, the organization should track economic performance and reward its employees according to the criteria of achieving environmental goals [63]. Moreover, improving environmental performance will enhance the economic performance of the firm [64]. Linking the green compensation with environmental issues will enhance employee’s commitment and efforts to green goals [65].

Performance management is a process in which the employees are encouraged to enhance their professional skills to better achieve organizational goals [66]. The emergence of environmental management and its impact on global business strategies is influenced by the phenomena of “greening” [67]. The most crucial aspect of performance management is performance appraisal; effective performance appraisals prove to support continuous improvement in the organization’s outcomes. Monetary and non-monetary rewards are powerful tools to help environmental management [68]. Among the human resource practices, incentives and appraisals are one of the primary means of connecting the interests of both organization and employees. Compensation can motivate the employees to exert their maximum potential and achieve more environmental goals. Paying incentives or rewards, encouraging managerial support to sustain business processes and increase the attention of the top-level management and employees to achieve the environmental objectives [65,69]. Based on these discussions, the following hypothesis of the study is proposed:

**Hypothesis** **4** **(H4).** *Green performance management and compensation significantly mediate the relationship between green hiring and environmental performance.*

**Hypothesis** **5** **(H5).** *Green performance management and compensation significantly mediate the relationship between green hiring and economic performance.*

Green protocols are implemented in all operational, functional departments of the organizations to meet the increasing demand for corporate social responsibility [70]. Particularly, the social performance is linked with the CSR strategies of the firm. Green human resource strategies play a vital role in achieving social performance [71]. Similarly, the “Go green movement” had created green jobs in the organization [72]. Currently, firms have started linking performance management and appraisal system with sustainable goals. Ultimately, achieving these sustainable goals tends to improve and enhance the social performance of the firm [73]. Green programs and strategies help human resources to improve social responsibility concepts between the workforce. Thus, green human resource management plays a vital role in boosting social performance in an organization [74]. Therefore, based on the above-mentioned discussions, the following hypothesis of the study is proposed:

**Hypothesis** **6** **(H6).** *Green performance management and compensation significantly mediate the relationship between green hiring and social performance*.

**Figure 1:**
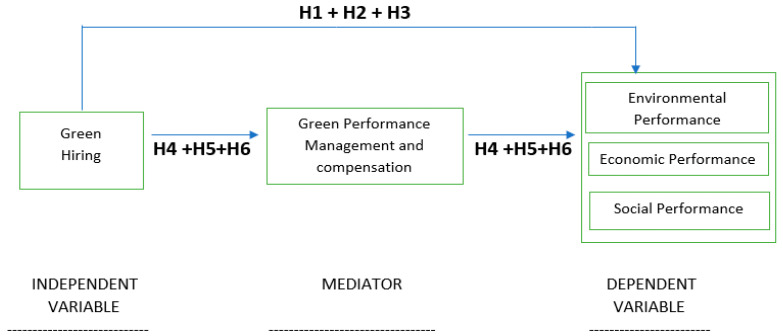
Conceptual framework.

The conceptual framework of the study is represented in Figure 1.

## 3. Methods and Research Design

This study focuses on the response of hospital staff towards green hiring and the sustainable performance of healthcare organizations. The unit of analysis is individual in this study. The target population of this study comprises HR staff and non-HR staff, positioned at different levels of corporate hierarchy (top, middle, low) of public as well as private hospitals of a developing country.

The research instrument was adapted from a recent study by Othman [11]. Primary data have been collected by the detailed and comprehensive research questionnaire; used to measure green hiring and sustainability of healthcare organizations. The sustainability of health organizations has been assessed by measuring environmental performance, economic performance, and social performance of public and private hospitals. The Likert scale has been used to record and elicit the responses of staff working in hospitals. A measurement scale (‘strongly disagree’ to ‘strongly agree’ ranging values from 1 to 5) is used to assess the level of agreement of respondents with the respective measures. Demographic details are also asked from respondents.

A self-administered questionnaire was distributed online and physically. An online questionnaire was developed with the help of Google forms. Data was collected through online surveys because data collection was a big challenge in times of a global pandemic. The current study was conducted during the COVID-19 pandemic situation, so it was difficult to meet staff working in hospitals for data collection. In total, 300 questionnaires were distributed. Out of which 200 questionnaires were distributed online and 100 were distributed physically and in total 173 were returned filled. After data collection, responses of staff were scrutinized and eight incomplete responses from online surveys and five incomplete responses from the physical survey were screened out and left with 160 complete responses. Therefore, the response rate was approximately 53%. Moreover, the minimum sample size of this study was assessed with the help of 5–10 times the number of items on the scale [75]. Therefore, the number of the items is 29, and the minimum sample size for the study will be 145 (29 * 5 =145). Moreover, Rascoe suggested the rule of thumb, when the population of the study is unknown then an appropriate sample size larger than 30 and smaller than 500 is appropriate for consideration [76]. Therefore, 160 responses in the study are considered to be appropriate for further analysis.

A multistage random sampling technique was used to study the green hiring process and sustainability of hospitals as it helps to provide more authentic and clear results. All hospitals were broadly classified into two categories—i.e., public and private. From all public and private hospitals, five hospitals were randomly selected. The staff of these hospitals was categorized into HR staff and non-HR staff, which also includes doctors. The healthcare staff was further divided based on the level of corporate hierarchy—i.e., top-level, middle level, and lower-level staff. Approximately, 43.75% and 56.25% responses were collected from private and public hospitals, respectively. The final sample comprised 61.3% non-HR staff and 38.8% HR staff. In addition to that, the highest percentage of respondents—i.e., 54.4% belongs to the middle level of the corporate hierarchy—while the lowest percentage (17.5%) belongs to respondents positioned at the lower level of the corporate hierarchy.

Data cleaning, data coding, and data screening are performed with the help of SPSS. SPSS is also used to analyze demographic profiles and descriptive statistics. Partial least square–structural equation modeling is used for hypothesis testing and to analyze the structural and measurement model of the study.

## 4. Results

Percentages and frequencies for each demographic characteristic were analyzed, along with their respective histograms, as the pictorial representation of data makes it more evident and more meaningful. Table 1 represents the demographic statistics of the respondents through the frequencies and percentages (n = 160). There are 55% female and 45% male respondents in the research study. 55% of participants are from private, and 45% are from public sector hospitals. Approximately 69% of the sample belongs to the urban background, and 31% are from rural families. The most dominant age group in this research is 20–30, and the most negligible percentage is the 50+ age group. Most of the people in the sample belong to non-HR positions, i.e., management and administrative staff at the hospital. The majority of the people belong to the middle level of the corporate hierarchy, as 54.4% of responses were taken from the middle-level staff, 28% were from the top level, and the remaining 17.5% of participants belonged to the functional group. The demographic profile helps to generalize the study results and better understand the respondent’s characteristics.

To get a better and clear image of those who were surveyed for this study, Table 2 is attached below representing the name of public and private hospitals and the number of questionnaires distributed and collected from them.

### 4.1. Measurement Model Assessment

The first step in measurement model estimation is to measure internal consistency reliability. To assess the internal consistency of the measurement model, the construct reliability and validity were measured through Cronbach alpha and composite reliability. Cronbach alpha ranges from 0 to 1; the higher and closer the value to 1 indicates a higher level of internal consistency. It ultimately leads to reliability [77]. The composite reliability is considered a more appropriate approach to measure internal consistency, and the values greater than 0.7 are considered substantial. Table 3 shows that Cronbach alpha values of all the relative constructs are more significant than 0.7, and the composite reliability of all constructs also exceeds 0.7, which shows the internal consistency of the construct. It reflects that all the indicators of each construct show consistency in measuring that construct.

Convergent validity is the measure that tests whether a measure correlates positively with the alternative measure of the same construct. To evaluate and assess the concurrent validity, AVE (average variance extracted) has been considered. The factor loading greater than 0.708 indicates that the latent variable can explain the indicator’s at least 50% variance. At the same time, the AVE should exceed 0.5 (50%) [78]. So, All the AVE values are more significant than 0.5 (50%), which means convergent validity is established.

**Figure 2:**
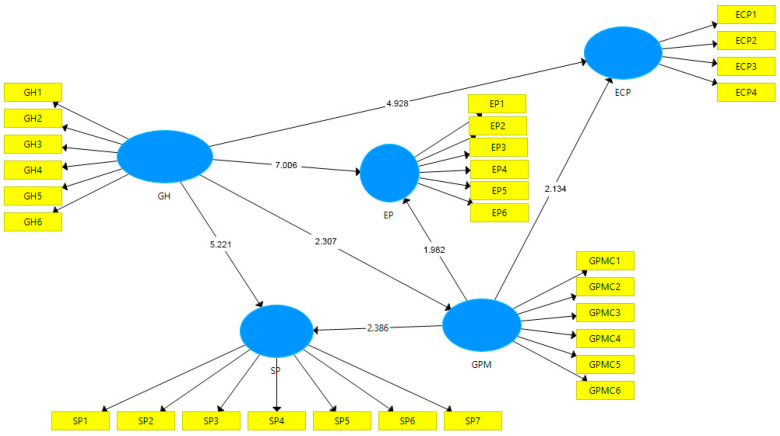
SEM model of the study.

To assess the extent to which a construct is distinct from other constructs. It implies that a construct is genuinely different and unique. To evaluate the discriminant validity, we consider Fornell and Larcker Criterion. Fornell and Larcker compare the square root of the AVE figures with the correlations of the latent variables. The square root of AVE should exceed the correlation of the other constructs [79]. The Fornell and Larcker criterion reflects the degree of shared variance between the construct of the model. Table 4 reflects that green performance management (GPMC) has the highest value, 0.811. From the importance of the F-L criterion, it is evident that these values are significant, and therefore discriminant validity is established. Whereas, Figure 2 represents the SEM model of the study.

### 4.2. Structural Model Assessment

In the first step of the structural model assessment, VIF values were evaluated to review the collinearity issues. All the values of VIF are in the range of 1.672 to 2.317, i.e., there is no collinearity issue. In the second step, the path coefficient was evaluated to test the hypothesized relationship. Path coefficient is the coefficient linking construct in the structural model. It indicates the strength of the relationship or represents the hypothesized relationship. It ranges from −1 to +1. The closer the value to +1, it suggests a strong positive relationship, and a value closer to −1 indicates a strong negative relationship. The values near zero were not considered significant. PLS algorithm runs to find out the path coefficients, with the help of a table created and the graphical representation through a bar chart that clearly shows the relationships and their strength. Followed by the algorithm is bootstrapping, which was done to determine the significance of the path coefficients. The importance of the path coefficients is determined using the *p*-value and *t* value for each. We have considered 1.96 (significant level 5%) as the practical value for the *t* value, whereas the considerable value for *p* was taken as 0.05, i.e., 5%. It means all the values below 1.96 for *t*-value were not considered significant, whereas the values above 0.05 are considered non-significant. Table 5 depicts the path coefficients and their significance.

The path coefficient value of green hiring (GH) → environmental performance (EP) is 0.430, whereas for the relationship of green hiring (GH) → economic performance (ECP) is 0.347. The relationship between green hiring (GH) → social performance (SP) has the lowest value, i.e., 0.326. Whereas the *t* statistics for the relationship green hiring (GH) → environmental performance (ECP) is 7.006 (*t* > 1.96), more than the critical level, and the *p*-value for this path is also significant (*p* = 0.000). The *t* statistics for the relationship green hiring (GH) → economic performance (ECP) is 4.928 (*t* > 1.96), more than the critical level, and the *p*-value for this path is also significant (*p* = 0.000). Similarly, *t* statistics and *p*-value for the relationship of green hiring (GH) → social performance (SP) are 5.221(*t* > 1.96), and 0.000, respectively.

Coefficient of determination (R^2^ Value) is the next step in the evaluation of the structural model. The R square was used to measure the variance explained, produced in the endogenous variable by the exogenous variable. The range of R square lies between 0 to 1. Table 6 shows that the R square value of the ECP is 0.456, which is considered a weak to moderate impact, and the value of EP is 0.710, which is considered a strong impact. Whereas SP has an R^2^ value of 0.569, consider a moderate effect. The model is also considered parsimonious because there is a minor difference between the values of R square and R square adjusted.

### 4.3. Mediation Analysis

There are three indirect effects in this study: (1) GH → GPMC → ECP, (2) GH → GPMC → EP, and (3) GH → GPMC → SP. These suggested indirect relationships and their significance are represented in Table 7. The path coefficient value of GH → GPMC → ECP is 0.457, and the *t* statistics value is 3.522; more than a significant level (*t* > 1.96). Whereas the *p*-value is also significant. (0.000). The second indirect effect of the study is GH → GPMC → EP. The path coefficient value is 0.478, and similarly, the *t* statistics for this relationship is 3.698, more than the critical level (*t* > 1.96). Similarly, the third indirect effect path coefficient value is 0.429, and the *p*-value, *t* statistics are significant. Hence, mediating relationships in all three paths are considered significant.

## 5. Discussion

The current study investigated the impact of GHRM practice—i.e., green hiring—on environmental, economic, and social performance. In addition, this study also analyzed the mediating role of green performance management and compensation between green hiring and sustainable implementation of the firm.

Based on the current study outcomes, green hiring has a positive and significant impact on environmental performance. These outcomes are consistent with past research. It supports H1 as Masri & Jaaron (2017) highlighted that green hiring plays a very influential role in environmental performance [26,27,28]. Roscoe et al. (2019) explored that green human resources management practices positively influence ecological performance [80].

The current study findings also showed that green hiring has a positive and significant impact on economic performance. A past survey by Wagner depicted that implementation of GHRM practices increased the firm’s sustainable performance and explored the positive and significant impact of GHRM practices on the firm’s economic performance [54,55,56]. It is consistent with the findings of the current study and supports H2. The recent study also explored that green hiring has a positive and significant impact on social performance. This finding is consistent with Al Kerdawy’s (2019) past research, which suggested that GHRM practices positively correlate with corporate social responsibilities in the firm [81]. It supports H3 of the current study.

The current study has investigated that green performance management and compensation mediate the relationship between green hiring and environmental performance. In past research, Guerci et al. (2016) studied that green performance management and compensation mediates the relationship between customer pressure and ecological understanding [82]. The critical contribution of the current study relates to the mediating role of green performance management and settlement on the green hiring–environmental performance relationship. In other words, we can say that green performance management and compensation successfully allow firms to get better environmental performance by retaining those employees who are knowledgeable in ecology [21,35,43,57].

The current study analyzed that green performance management and compensation mediates the relationship between green hiring and social performance. In recent past research, Maas highlighted that providing quantitative balance to employees increases the corporate social responsibilities in employees, which leads to better social performance of the firm [83]. The critical contribution of the current study relates to the mediating role of green performance management and compensation between the green and hiring social performance relationship. In other words, we can say that green performance management and compensation successfully allow firms to get better social performance by recurring those employees knowing environmental aspects. The current study also revealed that green performance management and compensation mediates the relationship between green hiring and economic performance. Thus, the critical contribution of the recent research relates to the mediating role of green performance management and settlement on the green hiring–economic performance relationship. In other words, we can say that green performance management and compensation successfully allow firms to get better economic performance by recurring those employees knowing environmental aspects [60,68,71].

Outcomes of this study have revealed a good understanding of GHRM practices and the sustainability of firms. A contrast study of research carried out in the same area has been studied to build a better view and support the results achieved in the current study. Following are some discussions based on developing countries’ context exclusively to compare and evaluate the implementation of the GHRM concept in an underdeveloped country. Research conducted in the underdeveloped country investigates the impact of GHRM practices on the organization’s growth and shows a positive relationship between the implementation of GHRM practices and the organization’s prosperity. This past research has been conducted in the manufacturing industries; it supports the idea that the more is the GHRM practices introduced in different sectors, the more positive impact they will bring to the economy [84]. This conclusion is similar to the current research; the greater the implementation of GHRM practice—i.e., green hiring in the organization—the more the greening activities will be done, consequently achieving greater economic, social, and environmental sustainability.

Sadia Cheema, A.T. (2015) encouraged the effective use of green strategies in SME organizations. This past study has suggested introducing and focusing on implementing GHRM practices in organizations because of its ever-increasing demand. It highlighted the significance of GHRM practices as its benefits can be reaped in the long run with the completion of several organizational goals. This past study supports the present research findings, as it also promotes the usage of green operations and focuses on compliance with environmental standards yields a prosperous economy, safe environment, and a better community [85].

It can be seen from the findings of our research that there a positive relationship exists among the implementation of GHRM practice—i.e., green hiring—and attaining environmental, economic, and social sustainability across the public and private hospitals. The hospital industry has started the increased usage of green activities, values, and reward systems. These have led to more careful and efficient use of resources and a more protected ecological perspective. Many past studies have supported the fact that through green activities, the healthcare sector leads to a sustainable environment in all phases, including environmental, social, and economic perspectives. Another study suggested that organizations achieve sustainability through GHRM practices and highlighted that CSR is one vital field to create a positive image in society [86].

Greening is an effective and influential business strategy, which many organizations in all sectors adopt. This concept is widespread across the globe and is at the infancy level in developing countries too. The organizations have started implementing these strategies and have realized their importance. They have also started aligning their goals in this direction. Hospitals of developing countries have started focusing on promoting green hiring and compliance with environmental standards. Green evaluation and assessments are promoted; the employees are punished or rewarded based on their adherence to the environmental values followed in the organization. The job descriptions are designed based on green roles and responsibilities, and they recruit those employees who are motivated to work for the betterment of the environment [11,23,69].

It has been suggested that there is increased attention in taking initiatives for environmental and economic protection. Healthcare organizations have taken a keen interest in reducing the negative impacts on the community. There is increased attention towards recycling, waste management, and using green products for the organization. Efficient and careful use of resources is promoted in organizations.

## 6. Conclusions

The present study explores the impact of green hiring in achieving social, economic, and environmental sustainability in public and private hospitals of developing countries. The findings of the current study support the hypothesis that implementation of GHRM practice—i.e., green hiring has a positive and significant impact on the social, economic, and environmental sustainability of healthcare organizations. This study also analyzes that green performance management and compensation significantly mediates the relationship between green hiring and sustainable performance of healthcare organizations. The present study added to the evidence suggesting that that commitment towards implementing GHRM practices like green hiring will lead to increased environmental, economic, and social sustainability. Green performance management and compensation successfully allow firms to get more sustainable performance by recurring those employees knowing environmental aspects as greening is the need of the age to conserve the resources, preserve the ecology, and protect the community in the long run.

## 7. Implications, Limitations, and Recommendations

Health care organizations face greater pressure from customers and stakeholders to adopt green activities to protect the environment and preserve natural resources. Many studies have been conducted on GHRM practices and sustainability. However, the concept of GHRM is still evolving in developing countries, so there is a major need to dig deep into the concept of GHRM in different sectors.

Some past research has been conducted in one healthcare firm to explore the impact of GHRM practices on sustainable performance, but the current study has provided more generalized results by collecting the data from the public and private health care organizations. Therefore, policymakers can devise policies and strategies according to the need of public and private hospitals.

Another important contribution of this study is that it has explored the relationship and impact of green hiring on economic, social, and environmental performance. This study has supported past research by finding a positive relationship and a significant impact of green recruitment on sustainable performance. These findings provide information to both policymakers and managers to add environmental aspects to the job description, and employees should be selected on environmental standards.

Some past researchers have studied the impact of different GHRM practices on sustainability. However, the current study has used only one GHRM practice—i.e., green hiring—and studied whether green performance management and compensation mediates the relationship between green hiring and sustainability. It is the major contribution of this study as no past research has been conducted to study the mediating effect of green performance management and compensation in the relationship of green hiring–sustainable performance. This study provides information to the HR department of health care organizations to evaluate employee performance based on their remarkable ideas to improve, preserve, and save the environment. Employees should be punished for not meeting the environmental standards in the organizations. By applying these strategies, the social, economic, and environmental performance will be increased, and organizations can handle the customer stakeholder’s pressure.

Job seekers can get the idea of how important it is to be socially responsible and work in alignment with the environmental standards, as they will be hired, trained, and rewarded based on their degree of compliance with the green culture. This study is providing a sense of awareness among the public to be socially and environmentally aware.

Every research has some limitations, so while conducting this study, some limitations arose. The first limitation of this study is that the current study’s findings cannot be generalized because this study is limited to the public and private sectors of only developing countries. Restricted time, limited resources, and the global pandemic has made the study difficult and limit the exploration of many aspects. The concept of GHRM practices is still emerging in developing countries, so the staff of health care organizations lack the relevant knowledge; consequently, the outcomes of the current study have some generalization issue. Research questions of this study can be answered in future studies that can be carried out in other developing and developed countries to compare and test the current study’s findings. Few researchers have been conducted in service sectors, so the hypothesis of the current study can be tested in other service sectors rather than health care organizations. To dig deep into the concept of GHRM practices and their impact on sustainability, some moderation and mediation effects can be studied in future research. Future studies should answer how customer and stakeholder pressure can be handled by adopting different GHRM practices in firms.

## Figures and Tables

**Table 1:** Demographic profile of the respondents.

Items	Category	Distribution	
		Frequency	Percentage
Gender	Male	72	45%
	Female	88	55%
Sector	Public	72	45%
	Private	88	55%
Family Origin	Urban	110	68.8%
	Rural	50	31.3%
Age Bracket	20–30	78	48.8%
	30–40	62	38.8%
	40–50	18	11.3%
	50 above	2	1.3%
Job Designation	HR position	62	38.8%
	Non-HR position	98	61.3%
Level of Corporate Hierarchy	Top Level	45	28.1%
	Middle Level	87	54.4%
	Operational Level	28	17.5%

**Table 2:** References of questionnaire distributed and collected.

Hospitals	Questionnaires Collected	Questionnaires Distributed
General Hospital, Lahore	25	50
Children’s Hospital, Lahore	15	50
Farooq Hospital, Lahore	50	75
Sheikh Zahid Hospital, Lahore	50	75
National Hospital, Lahore	20	50
Total	160	300

**Table 3:** Construct reliability and validity.

Constructs	Cronbach’s Alpha	Composite Reliability	Average Variance Extracted (AVE)
ECP	0.797	0.791	0.526
EP	0.816	0.866	0.519
GH	0.895	0.920	0.657
GPMC	0.906	0.924	0.605
SP	0.814	0.860	0.623

**Table 4:** Fornell–Larker criterion.

Constructs	ECP	EP	GH	GPMC	SP
ECP	0.703				
EP	0.678	0.720			
GH	0.440	0.510	0.778		
GPMC	0.343	0.428	0.840	0.811	
SP	0.385	0.483	0.907	0.828	0.727

**Table 5:** Significance of path coefficients.

Suggested Paths	Original Sample (O)	Mean (M)	Standard Deviation (STDEV)	T Statistics	*p* Values
GH → ECP	0.347	0.356	0.070	4.928	0.000
GH → EP	0.430	0.438	0.061	7.006	0.000
GH → SP	0.326	0.337	0.062	5.221	0.000

**Table 6:** Coefficient of determination (R^2^ Value).

Endogenous Construct	R Square	R Square Adjusted
ECP	0.456	0.448
EP	0.710	0.708
SP	0.569	0.557

**Table 7:** Specific indirect effect.

Suggested Paths	Original Sample (O)	Mean (M)	Standard Deviation (STDEV)	T Statistics	*p* Values
GH → GPMC → ECP	0.457	0.468	0.130	3.522	0.000
GH → GPMC → EP	0.478	0.485	0.129	3.698	0.000
GH → GPMC → SP	0.429	0.446	0.116	3.688	0.000

## Data Availability

The data will be made available on request from the corresponding author.

## Abbreviations

GH	Green Hiring
ECP	Economic Performance
EP	Environmental Performance
SP	Social Performance
GPMC	Green Performance Management and Compensation
